# Peer Review of “COVID-19 Pneumonia Diagnosis Using Medical Images: Deep Learning-Based Transfer Learning Approach”

**DOI:** 10.2196/83234

**Published:** 2025-09-26

**Authors:** Ikenna Odezuligbo

**Affiliations:** 1 Creighton University, Omaha, NE, United States

**Keywords:** computer vision, COVID-19 pneumonia diagnosis, deep learning, transfer learning, medical imaging analysis

*This is the peer-review report for “COVID-19 Pneumonia Diagnosis Using Medical Images: Deep Learning-Based Transfer Learning Approach.”*

## Round 1 Review

### General Comments

This manuscript [1] describes a transfer-learning approach using pretrained convolutional neural networks (VGG16, VGG19, ResNet-50) for binary COVID-19 detection on chest X-ray and computed tomography images. Overall, it tackles a timely problem and reports high accuracy (>97%), but several methodological and reporting issues limit confidence in the findings and their reproducibility.

### Specific Comments

#### Major Comments

1. Lack of clinical validation: no in vivo or clinical ground-truth data are provided. The model’s >97% accuracy is based solely on public datasets; it’s unclear how it performs on real-world, heterogeneous clinical images.

2. Overfitting and hyperparameter tuning: identical performance across 5 hyperparameter settings for VGG16 suggests under- or overfitting. No learning curves or regularization impact analyses are shown to substantiate robustness claims.

3. Model comparison baseline: no comparison against simple baselines (eg, logistic regression on hand-crafted features) or recent literature benchmarks is provided, making it difficult to evaluate novelty and real gain.

#### Minor Comments

4. Repeated headings: “Integration into Mobile/Cloud-based Platform” appears twice in section 1; please consolidate.

5. Typographical and formatting errors: multiple sentences start without capitalization (eg, “we reviewing to the difference…”) and several references lack publication details (eg, [27,28] list only URLs).

## References

[1] Dharmik A (2025). COVID-19 pneumonia diagnosis using medical images: deep learning-based transfer learning approach. JMIRx Med.

